# Assessment of CA4+ Impact on Mechanical Properties of Articular Cartilage

**DOI:** 10.3390/ma18132943

**Published:** 2025-06-21

**Authors:** Matteo Berni, Simone Fantoni, Roberta Fognani, Paolo Cardarelli, Fabio Baruffaldi, Massimiliano Baleani

**Affiliations:** 1 Laboratorio di Tecnologia Medica, IRCCS Istituto Ortopedico Rizzoli, 40136 Bologna, Italy; 2 Division of Ferrara, Istituto Nazionale di Fisica Nucleare (INFN), 44122 Ferrara, Italy

**Keywords:** characterization of materials, soft tissue, mechanical properties, indentation, articular cartilage, cartilage degeneration, early diagnosis, X-ray imaging, contrast agent, contrast-enhanced imaging

## Abstract

X-ray imaging of articular cartilage could be a breakthrough for the early diagnosis of tissue degeneration. This approach relies on radiopaque contrast agents to enhance the visualization of soft tissues. The potential impact of contrast agents on the mechanical response of articular cartilage should be considered in the frame of both clinical and research applications. Attention has been drawn to a solution containing molecules with six iodine atoms and four positive charges (CA4+), which has been shown to improve the X-ray visibility of articular cartilage. This study aimed to determine the effects of a CA4+ solution on tissues’ mechanical properties. An experimental pipeline based on indentation tests was applied to paired samples of articular cartilage before and after the immersion in either CA4+ or phosphate-buffered saline solution, maintained at a temperature of 22 ± 2 °C, for 22 h to determine the differences in instantaneous, viscous, and equilibrium responses between the articular cartilage of the two groups. The 22 h immersion of articular cartilage in either CA4+ or phosphate-buffered saline solution had a significant detrimental effect on the overall response, including the instantaneous, viscous, and equilibrium responses, of the articular cartilage. However, this detrimental effect was greater with exposure to the CA4+ solution. Specifically, the articular cartilage was found to be less stiff in both the instantaneous response (approximately −25%) and the equilibrium response (approximately −38%). The softening effect could be attributable to an alteration of the interaction between the proteoglycans of articular cartilage, induced by the positive charges within the CA4+ contrast agent. Further investigations are needed to elucidate whether this hypothesized mechanism is reversible.

## 1. Introduction

Articular cartilage (AC) is an avascular and aneural tissue that covers the ends of opposing bone segments in diarthroses. Its unique mechanical properties, including the absorption of shocks and distribution of articular contact pressure during locomotion, derive from the interplay between phases, a solid matrix, and an interstitial fluid [1]. The main components of the solid matrix are bundles of collagen fibrils and proteoglycans (PGs), the most abundant of which are aggrecans. The three-dimensional arrangement and concentration of collagen and PGs define the extracellular matrix (ECM) [2], in which a small population of chondrocytes is embedded. The interstitial fluid is primarily composed of water and dissolved electrolytes (mainly Na^+^ ions) [3].

The response of such a complex structure to the sudden application of a localized pressure to the articular surface must be split into an instantaneous flow-independent and a flow-dependent viscoelastic response [2]. The instantaneous response is determined by the rapid localized deformation of the ECM, resulting in the localized tensioning of the collagen network in the superficial zone [4], reorientation of collagen network in the deep zone [5], and an increase in both the mutual repulsive force of glycosaminoglycans [3] and fluid pressure [6,7]. The localized increase in the fluid pressure drives interstitial fluid flow through the porous ECM and outward across the AC tissue over time. This process continues until the equilibrium between the applied load and the tissue reaction is reached, at which point the fluid flow ceases [8,9]. For the purposes of this study, it is also important to note that the fixed charge density (FCD) of the AC provides an osmotic pressure [10] counterbalanced by the restraining stress in the collagen network. The osmotic pressure, together with the mutual electrostatic repulsion between the PGs, determines the swelling of the AC tissue [2].

The degeneration of the complex structure of AC tissue, whatever the cause, can result in pain and loss of joint function, e.g., as occurs with the onset of osteoarthritis [11]. X-ray imaging is a diagnostic tool commonly used in the evaluation of a wide range of musculoskeletal disorders. However, the X-ray visibility of AC tissue is impaired by its low radiopacity. Two approaches have been proposed for enhancing the X-ray visualization of AC tissue: X-ray phase-contrast imaging and the use of contrast agents (CAs). Although different streams of X-ray phase imaging, e.g., propagation-based, diffraction-enhanced, edge-illumination, and grating interferometry, can be found in the literature [12,13,14,15], almost none have been successfully translated to clinical applications in the management of joint pathologies. Notably, a clinical system implemented with grating interferometry distinguished AC alterations in the metacarpophalangeal joints of patients affected by rheumatoid arthritis [16]. Despite these promising results, its effectiveness on other joints remains untested. On the other hand, CAs are selected for a specific imaging target depending on the tissue composition [17]. Limited to AC, the imaging outcome strictly depends on the net charge of the molecule composing the considered CA [18]. CAs can be categorized as ionic and non-ionic, based on whether the single molecule carries a net charge or is electrostatically neutral. Ionic CAs can be further subdivided into anionic and cationic, depending on whether the displayed charge is negative or positive, respectively. Non-ionic and anionic CAs have been extensively tested for AC X-ray imaging [19,20,21,22,23,24,25,26,27,28]. Despite their safety certified by regulatory bodies, clinical use of anionic and non-ionic CAs require considerable concentrations, due to the indirect correlation between their distribution and the AC composition cartilage (namely, a direct correlation with water content and an inverse correlation with PGs) [22,23,24,25]. An experimental cationic iodine-based CA, referred to as CA4+, was specifically developed to be electrostatically attracted by the PGs of cartilage tissue [29]. The preliminary results regarding the safety and toxicity of CA4+ are encouraging [29]. Additionally, significant correlations have been reported between the mechanical properties and CA4+-enhanced imaging features on different AC models and several X-ray imaging systems [29,30,31,32,33,34,35,36]. Indeed, CA4+ permeates within AC, in concentrations directly proportional to the PG content [29,31]. However, the exposure of AC tissue to CAs has already highlighted a significant effect on tissue mechanical properties [37]. Therefore, the impact of any new contrast agent, such as CA4+, on tissue mechanics should be thoroughly investigated before its use in clinical practice or biomechanical research, as alterations to the mechanical properties of AC may affect joint function or experimental outcomes.

The aim of this study was to investigate the possible effects of CA4+ on the mechanical behavior of AC tissue. Indentation tests were performed on AC samples exposed to CA4+ to determine the instantaneous, viscous, and equilibrium behavior of the tissue. The results were compared to the behavior of a control group, which underwent the same experimental protocol except for the exposure to the CA.

## 2. Materials and Methods

### 2.1. Preparation of Contrast Agent Solution

The CA used in the present work is CA4+ (5,50-[Malonylbis(azanediyl)]bis[N1,N3-bis(2-aminoethyl)-2,4,6-triiodoisophthalamide] chloride). CA4+ molecules include six iodine atoms each. They have the following characteristics: molecular weight mw = 1354 g/mol, iodine fraction per molecule f = 0.5084, net positive charge q = +4. Therefore, CA4+ molecules are electrostatically attracted to the negative FCD of PGs.

Synthesis of CA4+ salts was carried out according to the work of Stewart et al. [29]. CA4+ solution was prepared dissolving its salts in phosphate-buffered saline (PBS) solution (7.4 pH, Life Technologies Europe B.V., Bleiswijk, The Netherlands), to obtain a concentration of 10 mgI/mL. This value is lower than the 12 mgI/mL used in the aforementioned study. The rationale for this reduction is that a concentration of 10 mgI/mL has been proven effective in achieving the X-ray attenuation of AC [38]. Using a lower salt concentration to enhance AC radiopacity may reduce the risk of a possible impact on tissue and side effects in patients. The pH of the obtained solution was adjusted to a value of 7.4 by adding NaOH 4 M. The osmolality (osmometer TypM 10/25 µL, accuracy 1 mOsm/kg, Löser Messtechnik, Berlin, Germany) of the CA4+ solution was 419 mOsm/kg. For comparison, the osmolality of the PBS solution was 307 mOsm/kg.

### 2.2. Extraction of Osteochondral Tissue Samples

Parallelepiped shaped samples, approximatively 20 mm in thickness, were excised from fresh tibio-femoral surfaces of bovine stifle joints obtained from a local slaughterhouse within 24 h of slaughter. The parallelepiped shaped samples were taken from regions with different AC thicknesses to capture the full spectrum of thicknesses, and from both the femur and tibia to represent the entire joint. After excision, parallelepiped shaped samples were wrapped in gauzes soaked in PBS solution and frozen at T = −20 °C (first freezing cycle). After thawing in PBS at 4 °C for one hour, osteochondral (OC) cores—10 mm in diameter—were extracted from each excised parallelepiped shaped sample. Depending on its size, two or four OC cores were extracted from each parallelepiped shaped sample. OC cores were extracted by means of a computer numerically controlled milling machine (ProLight 2000, Light Machines Corporation, Manchester, NH, USA) equipped with a diamond-coated coring tool with a 10 mm inner diameter. The parallelepiped shaped sample was kept fully immersed in PBS solution at room temperature during the process. OC cores were extracted at points where the surface was as flat as possible, perpendicular to the local articular surface. Each OC core was cut on the trabecular bone side to adjust its height to approximately 10 mm. Therefore, each OC core included AC tissue, subchondral, and trabecular bone. OC cores from adjacent locations were paired. A total of 36 matched pairs of OC cores were extracted. For each pair of OC cores, one was randomly assigned to the CA4+ treated (contrast-enhanced CE) group while the other was assigned to the PBS-treated (Control) group. After harvesting, each OC core was enveloped in PBS-soaked gauze and frozen at T = −20 °C until Parallelepiped indentation test (second freezing cycle).

### 2.3. Indentation Tests

Subgroups of six OC cores were thawed in PBS solution at T = 4 °C overnight. The AC thickness was measured prior to testing. Each OC core was blotted from any excess of PBS solution and placed in a custom-made polymethyl methacrylate sample holder. Four planar images of each OC core were acquired via X-ray µCT system (SkyScan 1072, SkyScan, Aartselaar, Belgium), each after a 90–rotation of the sample (X-ray tube voltage = 50 kV, current = 197 µA, 1-cm Al filter, pixel size = 11.5 µm, exposure time = 5.9 s). AC thickness was determined using a custom-made MATLAB code (MATLAB version R2022b, MathWorks, Natick, MA, USA), according to the following steps: (i) preliminary crop of planar images to exclude residual portion of the sample holder; (ii) differentiation of AC layer from mineralized tissue and air; (iii) binarization of AC layer; (iv) exclusion of possible spurious pixels; (v) counting of the number of pixels within the AC layer in the vertical direction. The counting was limited to a 10 pixel-thick peripheral annulus of the AC layer to avoid the inevitable overlap caused by projecting the 3D curved layer onto the planar image, which could introduce errors to the thickness calculation; (vi) averaging the calculated values over the four planar images.

The six OC cores were then placed in a custom-made polyacetal sample holder with six cavities, with an inner diameter of 10 mm and depth of 20 mm. Before placing the OC cores in the cavities, a small amount of polymethyl methacrylate was poured into the bottom of each cavity to constrain the core on the trabecular bone side, and a silicone-based grease was applied to the lateral surface of AC. It has been proven that a small continuous amount of grease around the entire circumference prevents any leakage of aqueous solution into the small gap between the edge of AC and the inner surface of the cylindrical cavity [38]. Then, 0.5 mL of PBS solution was added to each cavity, i.e., on the top surface of the OC cores, to keep the AC wet. The sample holder was mounted on the X-Y motorized table of the testing machine (Mach-1 V500css, Biomomentum Inc., Laval, QC, Canada). Each OC core underwent the following procedure: (i) a preliminary indentation to precondition the AC tissue, (ii) three indentations, each performed after a resting period of 40 min, (iii) resting for 22 h at a temperature of 22 ± 2 °C, to ensure that diffusion equilibrium had been reached [38] while keeping the sample mounted on the testing machine to ensure that the indentation test is performed in the same position, after replacing the PBS solution with 0.5 mL of CA4+ (CE group) or fresh PBS solution (Control group), (iv) an additional preliminary indentation, (v) three indentations, each performed after a resting period of 40 min (see Figure S1 in the Supplementary Materials Section). This two-groups experimental design was necessary to distinguish the effect of CA4+ from any modifications in the mechanical response of the AC due to tissue alterations over time. The entire process for each subgroup of six OC cores took 30 h, during which the OC cores were kept at room temperature [39].

The indentation test involved applying a 6 mm spherical indenter perpendicular to the articular surface of the OC core (see Figure S2 in the Supplementary Materials Section) at deformation rate of 0.15 s^−1^, i.e., 15%/s. To ensure consistent preloading at the start of the indentation test, the initial contact between the indenter and AC was defined using a load-based criterion, i.e., when the load reached 70 mN. The test was conducted to achieve a maximum nominal deformation equal to 15% of AC thickness at the center of the OC core [40]. The nominal deformation was maintained for 300 s [41] to monitor the stress response over time (relaxation test). A pilot study had previously been carried out to verify that this duration would result in a negligible load relaxation rate (<0.01 N/s) by the end of the period. The above reported protocol was used for both the preliminary indentation and the three indentations employed to determine the mechanical properties of AC (see Figure S1 in the Supplementary Materials Section). The instantaneous response of AC was evaluated in terms of maximum reaction load (S_0_) reached during indentation, and instantaneous elastic modulus (E_0_). S_0_ was measured experimentally by the multiple-axis load cell (accuracy 1% within the measurement range). E_0_ was calculated by fitting the load-displacement indentation curve to the Hayes model [42], by setting the Poisson’s ratio to 0.45 [43]. The viscous behavior of the AC was evaluated in terms of load decay, starting from the beginning of the test, and speed at which the equilibrium was reached. This behavior was quantified by the time constant (τ) and the stretching parameter (β) of a stretched exponential function [44] used to fit the load–time relaxation curve (see Appendix A). Such a function was implemented by minimizing the root mean square error through a custom-made MATLAB script. The fluid flow-independent property of AC, i.e., when all fluid flow has ceased, was evaluated in term of equilibrium modulus (E_eq_). E_eq_ was calculated using the Hayes equation, based on the load measured experimentally by the load cell at equilibrium, i.e., 300 s after the start of the indentation test, and considering the involved geometries [41]. In calculating E_eq_ of AC, a Poisson’s ratio of 0.3 was assumed [45,46]. All the values of the five parameters (S_0_, E_0_, τ, β, E_eq_) were normalized to the value measured in the first indentation test to determine the percentage change between subsequent indentation tests.

### 2.4. Statistical Analysis

Differences in AC thickness between the CE and Control groups were assessed using the Wilcoxon signed-rank test.

Differences in S_0_, E_0_, τ, β, or E_eq_ values measured during the first indentation between the CE and Control groups were also evaluated with the Wilcoxon signed-rank test.

Any upward or downward trend in S_0_, E_0_, τ, β, or E_eq_ values measured during the first three indentations, i.e., before 22 h of immersion in solution, or the second three indentations, i.e., after 22 h of immersion in solution, were assessed using the Friedman test.

The effect of 22 h immersion in solution on the mechanical behavior of the AC within each group was investigated by applying the Mann–Whitney signed-rank test to the normalized values of S_0_, E_0_, τ, β, or E_eq_, i.e., the changes in the values of the five parameters between the two series expressed as per cent of the initial value (percentage change). Finally, the effect of the CA4+ solution was analyzed by applying the Mann–Whitney test to compare the percentage changes between the CE and Control groups.

## 3. Results

The AC thickness values of OC cores for the CE group and Control group were in the range of 0.8–3.1 mm and 0.8–3.0 mm, respectively. No significant difference was found between the two groups (CE group median thickness 1.5 mm, Control group median thickness 1.4 mm, Wilcoxon signed-rank test: *p* = 0.82). By considering a sphere-plane Hertzian contact, the contact radius (*a*) was calculated to be in the range of 0.8–1.6 mm. Since the AC area affected by indentation extends to less than 3*a* [40]—which in our series was always smaller than the OC core radius—boundary effects were unlikely to influence the AC tissue response to indentation.

A total of 432 indentation tests were performed, neglecting the preconditioning procedure of AC tissue. The indentation procedure on the untreated AC tissue appeared to be repeatable, as evidenced by the coefficient of variation calculated for the five mechanical parameters (Table 1).

The mechanical response of the AC tissue of the 72 OC cores measured during the first indentation differed by up to an order of magnitude. The range of variability, including the minimum and maximum values, of the computed mechanical parameters are reported in the following: S_0_: 0.7–9.9 N; E_0_: 0.6–28.9 MPa; τ: 1.0–15.4 s; β: 0.326–0.657; E_eq_: 0.1–1.2 MPa. No difference was found between the two groups in the S_0_ (Wilcoxon signed-rank test: *p* = 0.24), E_0_ (Wilcoxon signed-rank test: *p* = 0.73), τ (Wilcoxon signed-rank test: *p* = 0.27), β (Wilcoxon signed-rank test: *p* = 0.85), and E_eq_ (Wilcoxon signed-rank test: *p* = 0.08) values measured during the first indentation.

**Figure 1:**
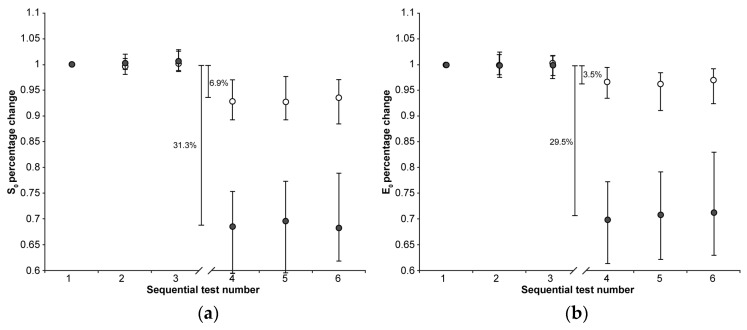
The median (markers), 75th percentile (upper edge of error bars), and 25th percentile (lower edge of error bars) of the percentage changes in S_0_ (**a**) and E_0_ values (**b**) before (1st, 2nd, and 3rd indentation test) and after (4th, 5th, and 6th indentation test) 22 h of immersion in solution are shown (grey markers: CE group; white markers: Control group). The values shown in the figure were calculated as the differences between the medians of the 108 values measured in the first three and the 108 values measured in the second set of indentation tests.

**Figure 2:**
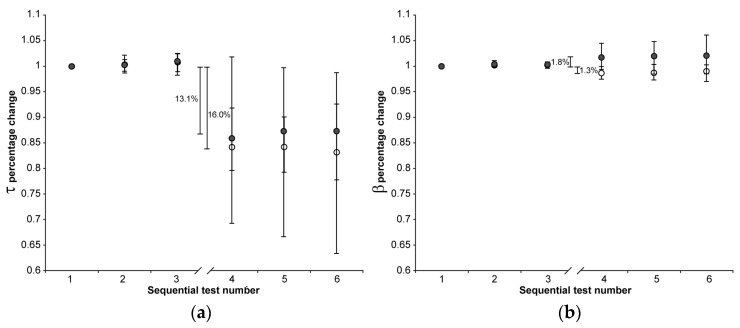
The median (markers), 75th percentile (upper edge of error bars), and 25th percentile (lower edge of error bars) of the percentage changes in τ (**a**) and β values (**b**) before (1st, 2nd, and 3rd indentation test) and after (4th, 5th, and 6th indentation test) 22 h of immersion in solution are shown (grey markers: CE group; white markers: Control group). The values shown in the figure were calculated as the differences between the medians of the 108 values measured in the first three and the 108 values measured in the second set of indentation tests.

**Figure 3:**
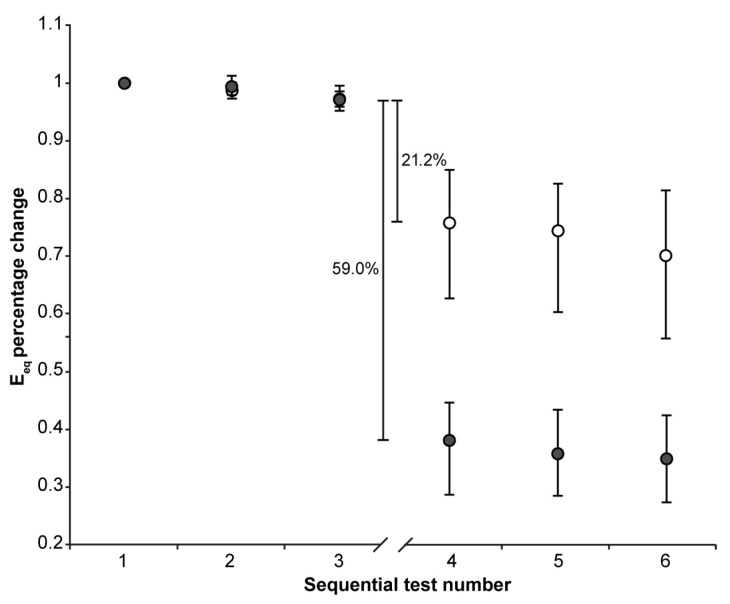
The median (markers), 75th percentile (upper edge of error bars), and 25th percentile (lower edge of error bars) of the percentage changes in Eeq values before (1st, 2nd, and 3rd indentation test) and after (4th, 5th, and 6th indentation test) 22 h of immersion in solution are shown (grey markers: CE group; white markers: Control group). The values shown in the figure were calculated as the differences between the medians of the 36 values measured in the third and the 36 values measured in the fourth set of indentation tests to exclude the effect of the downward trend.

No significant trends over test repetitions were found in the first three or the second three indentations in both the CE and Control group for all calculated parameters, except E_eq_:E_eq_ values showed a small, but systematic, downward trend in both the first three and the second three indentations regardless of the group (Friedman test: *p* < 0.001 in all four cases). The downward trend was similar for the two groups in the first three repetition (median reduction between the first and the third indentations about 3%), as was the reduction observed in the CE group in the second three indentations (median reduction between the fourth and the sixth indentations about 3%) The reduction observed in the Control group in the second three indentations was a few percentage points greater (median reduction between the fourth and sixth indentation about 8%). To exclude the effect of such a downward trend, the effect of CA4+ on E_eq_ was calculated as the differences between the medians of the 36 values of the third and fourth indentation sets. Normalized parameter trends across tests are shown in Figure 1, Figure 2 and Figure 3.

The 22 h immersion of AC in the CA4+ solution had the following significant effects (Mann–Whitney signed-rank test: *p* < 0.001 in all five cases): 31.3% reduction in S_0_, 29.5% reduction in E_0_, 13.1% reduction in τ, 1.8% increase in β, 59.0% reduction in E_eq_, with the latter calculated as the difference between the medians of the 3rd and 4th indentation tests.

On the other hand, keeping the AC immersed in the PBS solution had the following significant effects (Mann–Whitney signed-rank test: *p* < 0.001 in all five cases): 6.9% reduction in S_0_, 3.5% reduction in E_0_, 16.0% reduction in τ, 1.3% reduction in β, 21.2% reduction in E_eq_, with the latter calculated as the difference between the medians of the 3rd and 4th indentation tests.

**Figure 4:**
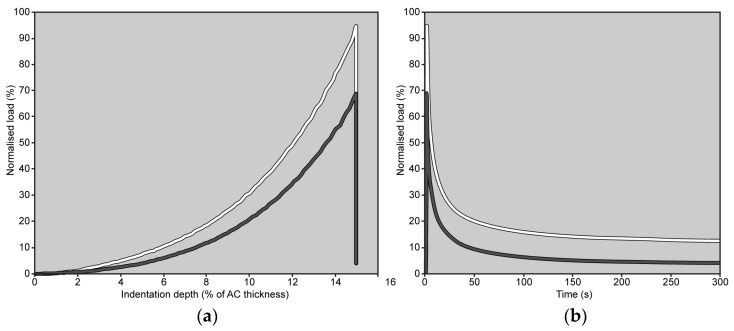
Load trends measured on matched paired OC cores (grey line: OC core immersed in CA4+ solution; white line: OC core immersed in PBS solution) during the 4th indentation are shown as a function of indentation depth, up to 15% of AC thickness (**a**), and as a function of time (**b**). The peak load is reached in 1 s. In both graphs, the load values have been normalized to the maximum load measured on the same OC core during the 1st indentation (note: if there were no alterations in the mechanical response of the AC, the peak load should have reached 100%).

When comparing the percentage changes between the two groups, immersion in CA4+ solution led to the following significant differences relative to immersion in PBS solution: a 24.4% reduction in S_0_ (Mann–Whitney: *p* < 0.001), a 26.0% reduction in E_0_ (Mann–Whitney: *p* < 0.001), 37.8% reduction in and E_eq_ (Mann–Whitney: *p* < 0.001), and a 3.1% increase in β (Mann–Whitney: *p* < 0.001). No significant difference was found in the τ values between the two groups (Mann–Whitney: *p* = 0.76). Figure 4 is representative of the response measured on matched paired OC cores after 22 h of immersion in CA4+ or PBS solution.

## 4. Discussion

The aim of the present study was to investigate possible alterations in the AC mechanical response related to the use of a cationic iodine-based contrast agent CA4+.

The design of this study attempted to account for the altered mechanical response of the AC resulting from simply managing the tissue. Indeed, immersion in PBS solution, maintained at a temperature of 22 ± 2 °C, for 22 h, alters the AC tissue response, though to a lesser extent than immersion in CA4+ solution. This result deserves a brief discussion before addressing the effect of CA4+ on the tissue. It has been found that immersion of AC in PBS solution, maintained at a temperature of 4 °C, for 6 days does not significantly alter the mechanical behavior of the tissue, while a significant effect has been found after 12 days [47]. It is also known that increasing the temperature of the solution increases the degradation rate [48]. Therefore, it is reasonable to assume that the temperature of 22 °C used in this study accelerated the process reported by Changor et al. [47]. On the other hand, it must also be considered that the sample size of this study, along with the statistical analysis method—which tracks variations in each specimen—allows for the detection of statistically significant, even small percentage changes, even if of small entities. This is supported by the E_eq_ variation found in all 72 OC cores across the first three repetitions (Figure 3), which show a small but systematic decrease in E_eq_. In any case, the design of this study includes the control group with the aim of isolating the effect of the CA4+ on the mechanical properties of AC.

The comparison between the data acquired from the two groups suggest that the exposure to CA4+ alters both the instantaneous and equilibrium response of AC to a localized compression (i.e., indentation). As both the responses do not imply any fluid flow, it can be inferred that the altered response is due to a modification in the ECM tridimensional arrangement and, thus, a reaction to an external impulse. PGs located within the ECM confer FCD to its structure. When the AC is immersed in saline solution, FCD determines the Donnan osmotic pressure, AC swelling, and, ultimately, the collagen framework pretension [49]. The key role of FCD in the compressive behavior of AC is therefore heavily influenced by both the concentration and type (i.e., valence) of dissolved cations [50]. Indeed, it has been demonstrated that AC immersed in a physiological concentration solution in which monovalent ions (Na^+^) were replaced by divalent ions (Ca^2+^) leads to a compaction of the PG layers as well as bridging between the opposing PG layers [49]. The compaction of the adjacent PG layers, and the subsequent softening of AC, is expected to be further increased after AC immersion in a solution with a higher ion concentration also containing quadrivalent cations [51]. This is the case of the interaction between the positively charged molecules of CA4+ and negatively charged PGs. As a result, the PG molecules crimp, decreasing their spatial extension and, thus, the pretension applied to collagen bundles. The direct consequence of such a phenomenon could be associated with a decrease in the fluid-independent mechanical response of AC, i.e., the instantaneous and equilibrium response [52]. The osmolality of the solution is also a contributing factor to the softening mechanism. Indeed, it has been demonstrated that the immersion of bovine AC in a hyperosmolar solution of anionic CA induces an overall softening of the tissue. Such an effect was attributed to a decrease in pressure between the tissue and the CA bath, along with diminished repulsive forces exerted by PGs. Furthermore, the immersion in this hypertonic CA solution determined a net water flow exiting the tissue. Overall, the tension among collagen fibers decreased, resulting in a reduced fluid pressure under loading [53]. Although in the present study, the difference in osmolality between the CA4+ solution and PBS (100 mOsm/kg) is smaller if compared to the aforementioned study (300 mOsm/kg), the described softening mechanism may have played a reduced, but still present, role.

On the other hand, the exposure to CA4+ seems to have a minimal effect on the viscous properties of AC tissue. Indeed, small variations—a slight increase in the value of the stretch parameter β—were observed in the shape of the decay, specifically the final phase of the relaxation when the pressure gradients within AC are reduced. It is worth noting that all other parameters—therefore, S_0_ and τ—being equal, a slight increase in the stretch parameter β results in a slightly steeper knee of the curve, leading to a shorter time to reach the horizontal asymptote (see Appendix A). However, not significant variations were observed in the initial transient, i.e., in the fast initial decrease in the curve of Figure 4b, perhaps due to the large scatter of this response. On the other hand, the initial transient depends on the pressure impulse produced by the indentation. As CA4+-treated AC exhibits smaller values of instantaneous modulus, and therefore lower pressure impulse, it cannot be excluded a priori that under similar pressure gradients, the initial transient might provide different results. Otherwise, the following can also be hypothesized (i) the distorted ECM, due to the exposure to CA4+, was less effective at counteracting the fluid flow through the AC layers than the undistorted ECM and/or (ii) the amount of fluid that must flow is reduced due to the immersion in a hypertonic CA4+ solution, which determined the aforementioned net water flow exiting the tissue. Whatever the cause, it seems to be a minor effect compared to the effect on the instantaneous and equilibrium response.

This study has some limitations. First, OC cores were extracted from bovine stifle joints. This choice was driven by the wide sample size of the paired OC cores—including intact AC—that needed to be collected in a reasonable timeframe. Opting for OC cores from human knees would have made it impossible to comply with the project timeline. Nevertheless, bovine AC presents similar viscoelastic trends to those of human AC [54]. Therefore, the findings retrieved by the present study can be extrapolated to human OC cores. Second, the AC of the OC cores exhibited highly variable mechanical behavior, despite the preliminary optimized indentation testing protocol. The high variability in the mechanical response has already been reported for AC [54,55,56,57,58] and was expected, given the criterion used for OC core extraction. However, by pairing OC cores, it was possible to obtain two groups with comparable mechanical properties. In addition, by using each OC core as its own reference, it was possible to calculate the relative variation in percentage points of the computed parameters, thus equalizing all OC cores regardless of the absolute mechanical response of their AC [40]. Third, all OC cores underwent two freeze–thaw cycles. Previous studies have suggested that freeze–thaw cycles may affect the mechanical properties of AC [59,60]. The design of this study involved the paired OC cores of the Control and CE group undergoing the same procedure. Thus, any effects of freeze–thaw cycles on the tissue mechanical properties should be equally reflected in both groups. Fourth, indentation tests were performed at a deformation rate of 0.15 s^−1^, i.e., 15%/s. Given that the AC thickness ranged from 0.8 to 3.1 mm, the corresponding indenter speed required to achieve this deformation rate was between 0.120 and 0.465 mm/s. To simulate impact loading conditions, these values should be increased by two orders of magnitude [61]. However, it has been demonstrated that the indenter speeds within the range used in this study ensure interstitial fluid support [62], therefore are appropriate for evaluating the AC instantaneous response. Additionally, the set deformation level of 15% is just one of the possible values that may occur in vivo. Nevertheless, this value is representative of the AC deformation under full body weight [63,64] and therefore is suitable for, and already adopted in, comparative biomechanical testing of the knee AC [65]. Fifth, possible effects due to differences in the osmolality of the CA4+ solution were not investigated. The investigation of modifications in osmolality would have introduced an additional source of variability, making unfeasible the study of all the possible combinations. However, the osmolality of the CA4+ solution used in the present study fell within the range reported in the literature and was selected due to the efficacy of such a formulation in enhancing the radiopacity of AC [23,29].

Despite these limitations, the acquired data enabled the evaluation, through comparison, of the effect of the exposure to CA4+ on the response of AC to indentation, highlighting significant alterations in both the instantaneous and equilibrium responses.

## 5. Conclusions

The exposure of AC to a CA4+ solution with an osmolarity of 419 mOsm/kg significantly alters both the instantaneous and equilibrium responses of AC to indentation. The effect appears to be a softening of the AC tissue, likely due to the interaction between the three-dimensional distribution of positive charges within CA4+ and the PGs in the ECM, which may alter the PG–PG interaction contributing to AC compressive behavior. Whether this hypothesized mechanism damages AC, or whether the effects of CA4+ exposure are reversible through articular lavage, remains to be demonstrated.

## Figures and Tables

**Table 1:** Distribution of the coefficient of variation for the five mechanical parameters across the first three indentation tests (untreated AC tissue).

Percentile	CE Group					Control Group				
	S_0_	E_0_	τ	β	E_eq_	S_0_	E_0_	τ	β	E_eq_
5^th^	0.3	0.6	0.4	0.1	0.5	0.6	0.4	0.2	0.1	0.5
25^th^	0.7	1.3	0.8	0.3	1.0	0.9	1.0	0.8	0.3	1.3
50^th^	1.3	1.6	1.3	0.5	2.1	1.6	1.9	1.4	0.4	2.2
75^th^	2.9	3.1	2.8	0.7	3.5	1.9	2.8	2.6	0.7	3.2
95^th^	6.4	8.0	5.4	1.3	8.5	3.3	4.1	8.0	1.5	4.2

**Figure A1:**
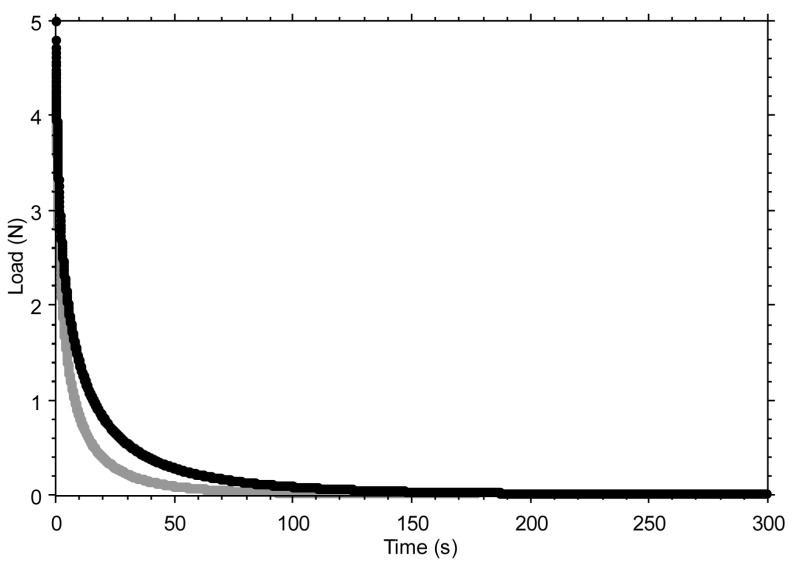
The effect of varying the time constant τ is shown by comparing τ = 6 s (black curve) and τ = 3 s (grey curve), while keeping all other parameters of the stretched exponential function unchanged (S_0_ = 5 N; β = 0.5; S_eq_ = 0 N). Note: The 50% reduction in the time constant was made for illustrative purposes only, to make the effect of the variation visible in the graph. S_eq_ was set to zero to better highlight how the final part of the curve tends toward a horizontal asymptote.

**Figure A2:**
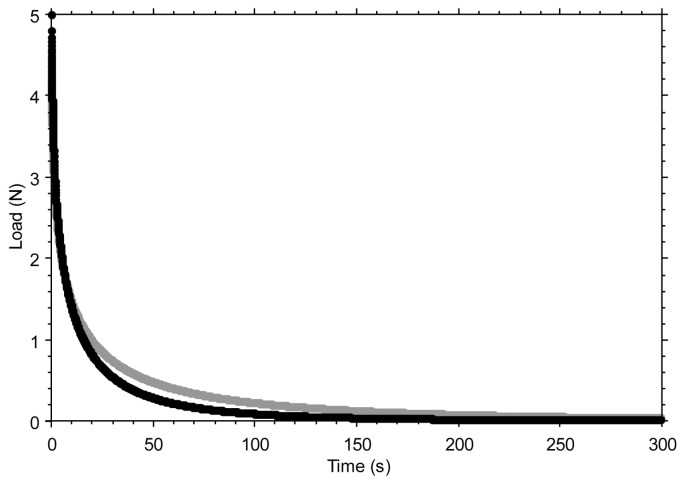
The effect of varying the stretching parameter β is shown by comparing β = 0.5 (black curve) and β = 0.4 (grey curve), while keeping all other parameters of the stretched exponential function unchanged (S_0_ = 5 N; τ = 6 s; S_eq_ = 0 N). Note: The 20% reduction in the stretching parameter was made for illustrative purposes only, to make the effect of the variation visible in the graph. S_eq_ was set to zero to better highlight how the final part of the curve tends toward a horizontal asymptote.

## Data Availability

The original contributions presented in this study are included in the article/Supplementary Material. Further inquiries can be directed to the corresponding author.

## Supplementary Materials

The following supporting information can be downloaded at: https://www.mdpi.com/article/10.3390/ma18132943/s1, Figure S1: flow chart showing the experimental design of this study. Figure S2: schematic of the indentation test. Annex S1: dataset of S_0_ values normalized to the value measured in the first indentation; Annex S2: dataset of E_0_ values normalized to the value measured in the first indentation; Annex S3: dataset of τ values normalized to the value measured in the first indentation; Annex S4: dataset of β values normalized to the value measured in the first indentation; Annex S5: dataset of E_eq_ values normalized to the value measured in the first indentation.

## Abbreviations

The following abbreviations are used in this manuscript:

AC	Articular cartilage
CA	Contrast agent
CE	Contrast-enhanced
ECM	Extracellular matrix
E_eq_	Equilibrium modulus
E_0_	Instantaneous elastic modulus
FCD	Fixed charge density
OC	Osteochondral
PBS	Phosphate-buffered saline
PG	Proteoglycan
S_0_	Maximum reaction load
β	Stretching parameter of the stretched exponential function
τ	Time constant of the stretched exponential function

## Appendix A

The stretched exponential model [66] is described by the function:

S(t) = (S_0_ − S_eq_) e^−(t/τ)β^ + S_eq_

where

S(t) is the load value at time t;
S_0_ is the maximum load value reached at the end of the indentation test;
τ is the time constant;
β is the stretching parameter (0 < β <1);
S_eq_ is the equilibrium load value reached at the end of the relaxation.

The load cell of the testing machine measured the load value S_0_ at the end of the indentation test, which was the initial load from which load relaxation began. It also recorded the load S(t) at a given time t and the equilibrium load S_eq_ at the end of the relaxation test. The two parameters τ and β were determined using a non-linear least-squares fitting routine.

Figure A1 shows the effect of variations in the time constant τ. A reduction in τ results in a larger value of 1/τ and, consequently, a faster drop in S(t) at the beginning of the relaxation test.

Figure A2 shows the effect of variations in the stretching parameter β. A reduction in β causes the curve to stretch more, thereby modifying the rate of decay, making it more gradual after the initial rapid drop.

## References

[1] Eschweiler J., Horn N., Rath B., Betsch M., Baroncini A., Tingart M., Migliorini F. (2021). The Biomechanics of Cartilage—An Overview. Life.

[2] Muir H. (1983). Proteoglycans as Organizers of the Intercellular Matrix. Biochem. Soc. Trans..

[3] Setton L.A., Zhu W., Mow V.C. (1993). The Biphasic Poroviscoelastic Behavior of Articular Cartilage: Role of the Surface Zone in Governing the Compressive Behavior. J. Biomech..

[4] Quiroga J.M.P., Wilson W., Ito K., Van Donkelaar C.C. (2017). Relative Contribution of Articular Cartilage’s Constitutive Components to Load Support Depending on Strain Rate. Biomech. Model. Mechanobiol..

[5] Hu J., Zheng K., Sherlock B.E., Zhong J., Mansfield J., Green E., Toms A.D., Winlove C.P., Chen J. (2025). Zonal Characteristics of Collagen Ultrastructure and Responses to Mechanical Loading in Articular Cartilage. Acta Biomater..

[6] Hayes W.C., Bodine A.J. (1978). Flow-Independent Viscoelastic Properties of Articular Cartilage Matrix. J. Biomech..

[7] Huang C.-Y., Mow V.C., Ateshian G.A. (2001). The Role of Flow-Independent Viscoelasticity in the Biphasic Tensile and Compressive Responses of Articular Cartilage. J. Biomech. Eng..

[8] Ateshian G.A. (2009). The Role of Interstitial Fluid Pressurization in Articular Cartilage Lubrication. J. Biomech..

[9] Mow V.C., Guo X.E. (2002). Mechano-Electrochemical Properties Of Articular Cartilage: Their Inhomogeneities and Anisotropies. Annu. Rev. Biomed. Eng..

[10] Lai W.M., Hou J.S., Mow V.C. (1991). A Triphasic Theory for the Swelling and Deformation Behaviors of Articular Cartilage. J. Biomech. Eng..

[11] Martel-Pelletier J. (2004). Pathophysiology of Osteoarthritis. Osteoarthr. Cartil..

[12] Horng A., Brun E., Mittone A., Gasilov S., Weber L., Geith T., Adam-Neumair S., Auweter S.D., Bravin A., Reiser M.F. (2014). Cartilage and Soft Tissue Imaging Using X-Rays: Propagation-Based Phase-Contrast Computed Tomography of the Human Knee in Comparison with Clinical Imaging Techniques and Histology. Investig. Radiol..

[13] Coan P., Bamberg F., Diemoz P.C., Bravin A., Timpert K., Mützel E., Raya J.G., Adam-Neumair S., Reiser M.F., Glaser C. (2010). Characterization of Osteoarthritic and Normal Human Patella Cartilage by Computed Tomography X-Ray Phase-Contrast Imaging: A Feasibility Study. Investig. Radiol..

[14] Marenzana M., Hagen C.K., Borges P.D.N., Endrizzi M., Szafraniec M.B., Vincent T.L., Rigon L., Arfelli F., Menk R.-H., Olivo A. (2014). Synchrotron- and Laboratory-Based X-Ray Phase-Contrast Imaging for Imaging Mouse Articular Cartilage in the Absence of Radiopaque Contrast Agents. Philos. Trans. A Math. Phys. Eng. Sci..

[15] Schulz G., Goetz C., Deyhle H., Mueller-Gerbl M., Zanette I., Zdora M.-C., Khimchenko A., Thalmann P., Rack A., Mueller B. Hierarchical Imaging of the Human Knee. Proceedings of the SPIE Optical Engineering + Applications—Development in X-Ray Tomography X.

[16] Momose A., Yashiro W., Kido K., Kiyohara J., Makifuchi C., Ito T., Nagatsuka S., Honda C., Noda D., Hattori T. (2014). X-Ray Phase Imaging: From Synchrotron to Hospital. Philos. Trans. A Math. Phys. Eng. Sci..

[17] Lusic H., Grinstaff M.W. (2013). X-Ray-Computed Tomography Contrast Agents. Chem. Rev..

[18] de Bournonville S., Vangrunderbeeck S., Kerckhofs G. (2019). Contrast-Enhanced MicroCT for Virtual 3D Anatomical Pathology of Biological Tissues: A Literature Review. Contrast Media Mol. Imaging.

[19] Aula A.S., Jurvelin J.S., Töyräs J. (2009). Simultaneous Computed Tomography of Articular Cartilage and Subchondral Bone. Osteoarthr. Cartil..

[20] Silvast T.S., Jurvelin J.S., Aula A.S., Lammi M.J., Töyräs J. (2009). Contrast Agent-Enhanced Computed Tomography of Articular Cartilage: Association with Tissue Composition and Properties. Acta Radiol..

[21] Silvast T.S., Jurvelin J.S., Lammi M.J., Töyräs J. (2009). pQCT Study on Diffusion and Equilibrium Distribution of Iodinated Anionic Contrast Agent in Human Articular Cartilage—Associations to Matrix Composition and Integrity. Osteoarthr. Cartil..

[22] Joshi N.S., Bansal P.N., Stewart R.C., Snyder B.D., Grinstaff M.W. (2009). Effect of Contrast Agent Charge on Visualization of Articular Cartilage Using Computed Tomography: Exploiting Electrostatic Interactions for Improved Sensitivity. J. Am. Chem. Soc..

[23] Bansal P.N., Joshi N.S., Entezari V., Malone B.C., Stewart R.C., Snyder B.D., Grinstaff M.W. (2011). Cationic Contrast Agents Improve Quantification of Glycosaminoglycan (GAG) Content by Contrast Enhanced CT Imaging of Cartilage. J. Orthop. Res..

[24] Bansal P.N., Stewart R.C., Entezari V., Snyder B.D., Grinstaff M.W. (2011). Contrast Agent Electrostatic Attraction Rather than Repulsion to Glycosaminoglycans Affords a Greater Contrast Uptake Ratio and Improved Quantitative CT Imaging in Cartilage. Osteoarthr. Cartil..

[25] Stewart R.C., Bansal P.N., Entezari V., Lusic H., Nazarian R.M., Snyder B.D., Grinstaff M.W. (2013). Contrast-Enhanced CT with a High-Affinity Cationic Contrast Agent for Imaging Ex Vivo Bovine, Intact Ex Vivo Rabbit, and in Vivo Rabbit Cartilage. Radiology.

[26] Rajendran K., Löbker C., Schon B.S., Bateman C.J., Younis R.A., de Ruiter N.J.A., Chernoglazov A.I., Ramyar M., Hooper G.J., Butler A.P.H. (2017). Quantitative Imaging of Excised Osteoarthritic Cartilage Using Spectral CT. Eur. Radiol..

[27] Flynn C., Hurtig M., Linden A. (2021). zur Anionic Contrast–Enhanced MicroCT Imaging Correlates with Biochemical and Histological Evaluations of Osteoarthritic Articular Cartilage. Cartilage.

[28] Baer K., Kieser S., Schon B., Rajendran K., Ten Harkel T., Ramyar M., Löbker C., Bateman C., Butler A., Raja A. (2021). Spectral CT Imaging of Human Osteoarthritic Cartilage via Quantitative Assessment of Glycosaminoglycan Content Using Multiple Contrast Agents. APL Bioeng..

[29] Stewart R.C., Patwa A.N., Lusic H., Freedman J.D., Wathier M., Snyder B.D., Guermazi A., Grinstaff M.W. (2017). Synthesis and Preclinical Characterization of a Cationic Iodinated Imaging Contrast Agent (CA4+) and Its Use for Quantitative Computed Tomography of Ex Vivo Human Hip Cartilage. J. Med. Chem..

[30] Lakin B.A., Grasso D.J., Shah S.S., Stewart R.C., Bansal P.N., Freedman J.D., Grinstaff M.W., Snyder B.D. (2013). Cationic Agent Contrast-Enhanced Computed Tomography Imaging of Cartilage Correlates with the Compressive Modulus and Coefficient of Friction. Osteoarthr. Cartil..

[31] Lakin B.A., Patel H., Holland C., Freedman J.D., Shelofsky J.S., Snyder B.D., Stok K.S., Grinstaff M.W. (2016). Contrast-Enhanced CT Using a Cationic Contrast Agent Enables Non-Destructive Assessment of the Biochemical and Biomechanical Properties of Mouse Tibial Plateau Cartilage. J. Orthop. Res..

[32] Bhattarai A., Honkanen J.T.J., Myller K.A.H., Prakash M., Korhonen M., Saukko A.E.A., Virén T., Joukainen A., Patwa A.N., Kröger H. (2018). Quantitative Dual Contrast CT Technique for Evaluation of Articular Cartilage Properties. Ann. Biomed. Eng..

[33] Honkanen M.K.M., Saukko A.E.A., Turunen M.J., Shaikh R., Prakash M., Lovric G., Joukainen A., Kröger H., Grinstaff M.W., Töyräs J. (2020). Synchrotron MicroCT Reveals the Potential of the Dual Contrast Technique for Quantitative Assessment of Human Articular Cartilage Composition. J. Orthop. Res..

[34] Freedman J.D., Ellis D.J., Lusic H., Varma G., Grant A.K., Lakin B.A., Snyder B.D., Grinstaff M.W. (2020). dGEMRIC and CECT Comparison of Cationic and Anionic Contrast Agents in Cadaveric Human Metacarpal Cartilage. J. Orthop. Res..

[35] Bhattarai A., Pouran B., Mäkelä J.T.A., Shaikh R., Honkanen M.K.M., Prakash M., Kröger H., Grinstaff M.W., Weinans H., Jurvelin J.S. (2020). Dual Contrast in Computed Tomography Allows Earlier Characterization of Articular Cartilage over Single Contrast. J. Orthop. Res..

[36] Paakkari P., Inkinen S.I., Honkanen M.K.M., Prakash M., Shaikh R., Nieminen M.T., Grinstaff M.W., Mäkelä J.T.A., Töyräs J., Honkanen J.T.J. (2021). Quantitative Dual Contrast Photon-Counting Computed Tomography for Assessment of Articular Cartilage Health. Sci. Rep..

[37] Pouran B., Arbabi V., Zadpoor A.A., Weinans H. (2016). Isolated Effects of External Bath Osmolality, Solute Concentration, and Electrical Charge on Solute Transport across Articular Cartilage. Med. Eng. Phys..

[38] Fantoni S., Gabucci I., Cardarelli P., Paternò G., Taibi A., Cristofori V., Trapella C., Bazzani A., Assenza M., Zanna Bonacorsi A. (2022). A Cationic Contrast Agent in X-Ray Imaging of Articular Cartilage: Pre-Clinical Evaluation of Diffusion and Attenuation Properties. Diagnostics.

[39] Jin H., Lewis J.L. (2004). Determination of Poisson’s Ratio of Articular Cartilage by Indentation Using Different-Sized Indenters. J. Biomech. Eng..

[40] Berni M., Erani P., Lopomo N.F., Baleani M. (2022). Optimization of In Situ Indentation Protocol to Map the Mechanical Properties of Articular Cartilage. Materials.

[41] Mohammadi A., Te Moller N.C.R., Ebrahimi M., Plomp S., Brommer H., Van Weeren P.R., Mäkelä J.T.A., Töyräs J., Korhonen R.K. (2022). Site- and Zone-Dependent Changes in Proteoglycan Content and Biomechanical Properties of Bluntly and Sharply Grooved Equine Articular Cartilage. Ann. Biomed. Eng..

[42] Hayes W.C., Keer L.M., Herrmann G., Mockros L.F. (1972). A Mathematical Analysis for Indentation Tests of Articular Cartilage. J. Biomech..

[43] Li G., Lopez O., Rubash H. (2001). Variability of a Three-Dimensional Finite Element Model Constructed Using Magnetic Resonance Images of a Knee for Joint Contact Stress Analysis. J. Biomech. Eng..

[44] Kumar R., Pierce D., Isaksen V., Davies C., Drogset J., Lilledahl M. (2018). Comparison of Compressive Stress-Relaxation Behavior in Osteoarthritic (ICRS Graded) Human Articular Cartilage. Int. J. Mol. Sci..

[45] Ebrahimi M., Ojanen S., Mohammadi A., Finnilä M.A., Joukainen A., Kröger H., Saarakkala S., Korhonen R.K., Tanska P. (2019). Elastic, Viscoelastic and Fibril-Reinforced Poroelastic Material Properties of Healthy and Osteoarthritic Human Tibial Cartilage. Ann. Biomed. Eng..

[46] Kiviranta P., Rieppo J., Korhonen R.K., Julkunen P., Töyräs J., Jurvelin J.S. (2006). Collagen Network Primarily Controls Poisson’s Ratio of Bovine Articular Cartilage in Compression. J. Orthop. Res..

[47] Changoor A., Fereydoonzad L., Yaroshinsky A., Buschmann M.D. (2010). Effects of Refrigeration and Freezing on the Electromechanical and Biomechanical Properties of Articular Cartilage. J. Biomech. Eng..

[48] Pereira-Lobato C., Echeverry-Rendón M., Fernández-Blázquez J.P., González C., LLorca J. (2024). Mechanical Properties, in Vitro Degradation and Cytocompatibility of Woven Textiles Manufactured from PLA/PCL Commingled Yarns. J. Mech. Behav. Biomed. Mater..

[49] Zimmerman B.K., Nims R.J., Chen A., Hung C.T., Ateshian G.A. (2021). Direct Osmotic Pressure Measurements in Articular Cartilage Demonstrate Nonideal and Concentration-Dependent Phenomena. J. Biomech. Eng..

[50] June R.K., Mejia K.L., Barone J.R., Fyhrie D.P. (2009). Cartilage Stress–Relaxation Is Affected by Both the Charge Concentration and Valence of Solution Cations. Osteoarthr. Cartil..

[51] Han L., Dean D., Mao P., Ortiz C., Grodzinsky A.J. (2007). Nanoscale Shear Deformation Mechanisms of Opposing Cartilage Aggrecan Macromolecules. Biophys. J..

[52] Lu X.L., Sun D.D.N., Guo X.E., Chen F.H., Lai W.M., Mow V.C. (2004). Indentation Determined Mechanoelectrochemical Properties and Fixed Charge Density of Articular Cartilage. Ann. Biomed. Eng..

[53] Turunen M.J., Töyräs J., Lammi M.J., Jurvelin J.S., Korhonen R.K. (2012). Hyperosmolaric Contrast Agents in Cartilage Tomography May Expose Cartilage to Overload-Induced Cell Death. J. Biomech..

[54] Temple D.K., Cederlund A.A., Lawless B.M., Aspden R.M., Espino D.M. (2016). Viscoelastic Properties of Human and Bovine Articular Cartilage: A Comparison of Frequency-Dependent Trends. BMC Musculoskelet. Disord..

[55] Li H., Li J., Yu S., Wu C., Zhang W. (2021). The Mechanical Properties of Tibiofemoral and Patellofemoral Articular Cartilage in Compression Depend on Anatomical Regions. Sci. Rep..

[56] Thambyah A., Nather A., Goh J. (2006). Mechanical Properties of Articular Cartilage Covered by the Meniscus. Osteoarthr. Cartil..

[57] Moshtagh P.R., Pouran B., Korthagen N.M., Zadpoor A.A., Weinans H. (2016). Guidelines for an Optimized Indentation Protocol for Measurement of Cartilage Stiffness: The Effects of Spatial Variation and Indentation Parameters. J. Biomech..

[58] Kabir W., Di Bella C., Choong P.F.M., O’Connell C.D. (2021). Assessment of Native Human Articular Cartilage: A Biomechanical Protocol. Cartilage.

[59] Peters A.E., Comerford E.J., Macaulay S., Bates K.T., Akhtar R. (2017). Micromechanical Properties of Canine Femoral Articular Cartilage Following Multiple Freeze-Thaw Cycles. J. Mech. Behav. Biomed. Mater..

[60] Qu C., Hirviniemi M., Tiitu V., Jurvelin J.S., Töyräs J., Lammi M.J. (2014). Effects of Freeze–Thaw Cycle with and without Proteolysis Inhibitors and Cryopreservant on the Biochemical and Biomechanical Properties of Articular Cartilage. Cartilage.

[61] Shepherd D.E.T., Seedhom B.B. (1997). A Technique for Measuring the Compressive Modulus of Articular Cartilage under Physiological Loading Rates with Preliminary Results. Proc. Inst. Mech. Eng. H.

[62] Bonnevie E.D., Baro V.J., Wang L., Burris D.L. (2012). Fluid Load Support during Localized Indentation of Cartilage with a Spherical Probe. J. Biomech..

[63] Hosseini A., Van De Velde S.K., Kozanek M., Gill T.J., Grodzinsky A.J., Rubash H.E., Li G. (2010). In-Vivo Time-Dependent Articular Cartilage Contact Behavior of the Tibiofemoral Joint. Osteoarthr. Cartil..

[64] Liu F., Kozanek M., Hosseini A., Van De Velde S.K., Gill T.J., Rubash H.E., Li G. (2010). In Vivo Tibiofemoral Cartilage Deformation during the Stance Phase of Gait. J. Biomech..

[65] Schad P., Wollenweber M., Thüring J., Schock J., Eschweiler J., Palm G., Radermacher K., Eckstein F., Prescher A., Kuhl C. (2020). Magnetic Resonance Imaging of Human Knee Joint Functionality under Variable Compressive In-Situ Loading and Axis Alignment. J. Mech. Behav. Biomed. Mater..

[66] Elton D.C. (2018). Stretched Exponential Relaxation. arXiv.

