# Supplementary figures and images for: Assessment of CA4+ Impact on Mechanical Properties of Articular Cartilage
Source: Materials (Basel). 2025 Jun 21;18(13):2943. doi: 10.3390/ma18132943 (PMC12250616; doi:10.3390/ma18132943)

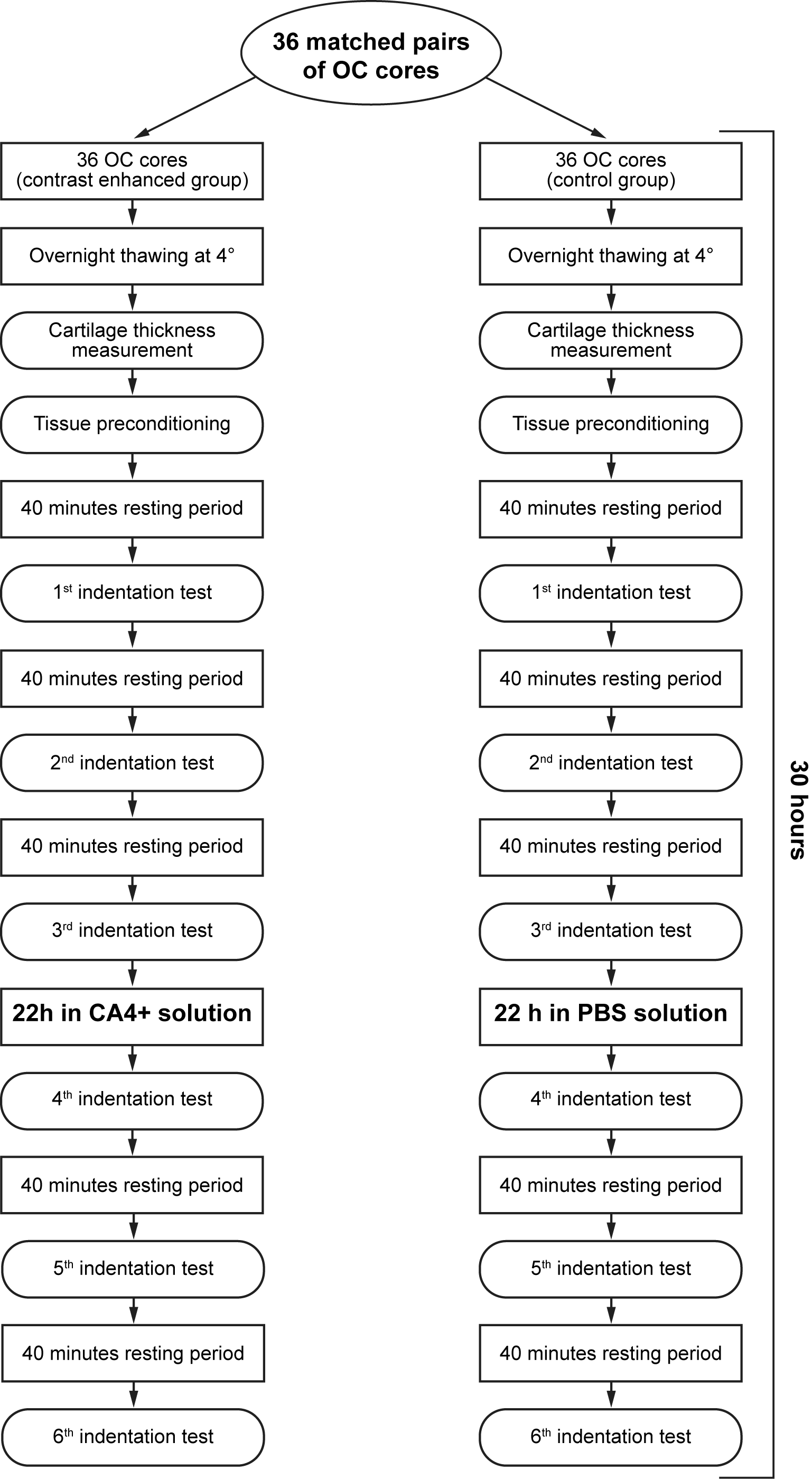

Supplement: Supplementary file 1 [file materials-18-02943-s001.zip › Figure S1.tif]

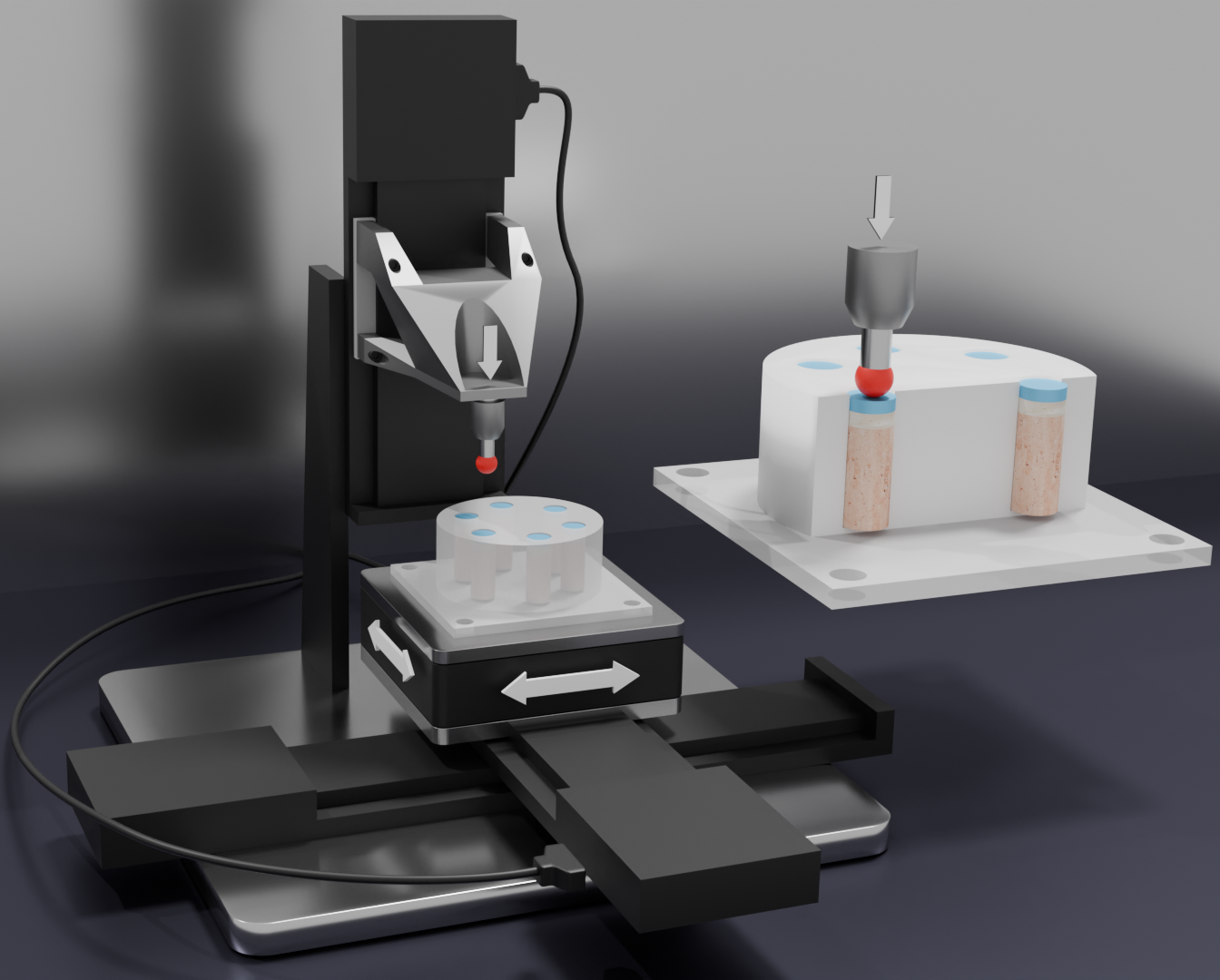

Supplement: Supplementary file 1 [file materials-18-02943-s001.zip › Figure S2.tif]
